# Supplementary material for: Photonic signal processor based on a Kerr microcomb for real-time video image processing
Source: Commun Eng. 2023 Dec 20;2:94. doi: 10.1038/s44172-023-00135-7 (PMC10956003; doi:10.1038/s44172-023-00135-7)
Supplement: Supplementary file 1 — Supplementary Information [file 44172_2023_135_MOESM1_ESM.pdf]

Supplementary Materials for

Photonic signal processor based on a Kerr microcomb for real time video image processing

Mengxi Tan,[1,2,3] Xingyuan Xu,[4] Andreas Boes,[3,5] Bill Corcoran,[6] Thach G. Nguyen,[3] Sai T. Chu,[7] Brent E. Little,[8] Roberto Morandotti,[9] Jiayang Wu,[2] Arnan Mitchell,[3] and David J. Moss [2,*]

[1]School of Electronic and Information Engineering, Beihang University, Beijing 100191, China.

[2]Optical Sciences Centre, Swinburne University of Technology, Hawthorn, VIC 3122, Australia

[3]School of Engineering, RMIT University, Melbourne, VIC 3001, Australia.

[4]State Key Laboratory of Information Photonics and Optical Communications, Beijing University of Posts and Telecommunications, Beijing, 100876, China.

[5]Institute for Photonics and Advanced Sensing (IPAS) and School of Electrical and Electronic Engineering, University of Adelaide, Adelaide, 5005 SA, Australia.

[6]Department of Electrical and Computer Systems Engineering, Monash University, Clayton, 3800 VIC, Australia.

[7]Department of Physics, City University of Hong Kong, Tat Chee Avenue, Hong Kong, China.

[8]Xi'an Institute of Optics and Precision Mechanics ofCAS, Xi'an, China.

[9]INRS-Énergie, Matériaux et Télécommunications, 1650 Boulevard Lionel-Boulet, Varennes, Québec, J3X 1S2, Canada.
Correspondence to: dmoss@swin.edu.au

This file includes:

Supplementary Text
Fig. S1 to S12
Table S1 to S2

1. Supplementary Note 1: Device characterization

The transmission and dispersion characterization of the MRR (which is fabricated on high-index doped silica glass platform) are experimentally measured via the MZI (Mach-Zehnder interferometer) method [Figures S1, S2]. Figure S1 shows the schematic diagram of the transmission and dispersion characterization measurement. The MRR transmission, from which Q factor and FSR values are determined, was measured via a tunable laser (Agilent 8164A) swept through wavelength from 1520 nm to 1620 nm and tuning range at a tuning rate of 20 nm/s. The MZI has a path length difference of about 45 m, making the measurement optical frequency sampling resolution approximately 4.6 MHz. The transmission spectra were derived from a

balanced photodetector (Thorlabs PDB460C-AC) and observed by the oscilloscope with a memory depth of 50 MPts. The measured and fitting results are shown in Figure S1 (g) and (h). The power fluctuations of the soliton crystal microcomb were measured over 120 hours (5 days), with the optical spectrum captured every 15 minutes (Figure S2 (a)).

2. Supplementary Note 2: Results

The first column of Figure S3 shows the simulated and experimental results of shaped comb spectra for fractional Hilbert transformer with tunable phase shift from 15° to 75°. A good match between the power of the measured comb lines (red solid lines for the positive tap coefficients and black lines for the negative tap coefficients) and the calculated ideal tap weights (gray dots) was obtained, indicating that the comb lines were successfully shaped. The second column of Figure S3 shows the shaped impulse response of the fractional Hilbert transform that has 75 wavelengths, or weights, in total. The 3rd to 4th column of Figure S3 presents the simulated (red dashed curves) and measured (black solid lines) transmission response magnitude and phase. The normalized frequency response and phase response of the fractional Hilbert transformer with tunable orders of 0.166, 0.333, 0.5, 0.667, 0.833, corresponding to phase shifts of 15°, 30°, 45°, 60°, 75° are shown. The last column of Figure S3 shows the images processed with the fractional Hilbert transformer. The phase shift is tunable which provides better flexibility in image enhancement [s3]. As can be seen, the edges in the image are enhanced, and the experimental results are consistent with the simulation results.

The simulated and experimental results of the shaped comb spectra, temporal impulse response, frequency response and processed image for fractional differentiation with tunable fractional orders from 0.1 to 0.9 are shown in Figure S4, with the shaped comb spectra in the first column. The wavelength channels for positive and negative taps were separately measured by the OSA. The optical power for each comb line closely matched the designed tap coefficients, verifying the success of our comb shaping procedure. The second column of Fig. S4 shows the shaped impulse response of the fractional differentiation. The measured |S21| curves denoted the power responses of the differentiator, with the operation bandwidth ranging from DC to 18 GHz. As shown in the 3rd and 4th column of Fig. S4, the range of the fractional orders resulted in a close match between the experimental and simulated results in terms of the slope coefficients of the power responses and the phase shift in the measured phase responses. The last column of Fig. S4 illustrates the processed images using the fractional differentiator, where the fractional order was tunable in order to provide better flexibility in edge detection.

Figure S5 shows the simulated and measured results of integral (ie., non-fractional) image processors including a Hilbert transformer with a 90° phase shift, an integrator, as well as first-order differentiator. As predicted in the last column of Fig. S5, the processed image with the Hilbert transformer can be used for edge enhancement, the integrator for motion blur, and the integral differentiator for edge detection. Figure S6 shows a reconfigurable analogue video image processor with operation bandwidth from 4.6 GHz to 36.6 GHz by varying the length of fibre (0.4 km vs 3.96 km) as well as the comb spacing (2-FSR to 8-FSR comb spacing).

We further verified the performance of the image processing function by calculating the root mean square errors (RMSE) between the measured waveforms and the theoretical results (Fig. S7). As shown in Table S1, the RMSEs of Hilbert transformer with tunable phase shift from 15° to 90° are 0.1045, 0.0876, 0.0622, 0.0586, 0.0807, 0.0599, respectively. As for the differentiator with tunable order from 0.1 to 1.0, the RMSEs are 0.0693, 0.0778, 0.0721, 0.0650, 0.0558, 0.0736, 0.0676, 0.0613, 0.0645, 0.0731, respectively. Finally, the RMSE of the integrator is 0.2595.

In addition to static image processing, our microcomb based RF photonic system also processed dynamic videos in real-time. Our results for real-time video processing are provided in supplementary Movie S2, while Supplementary Figures S8 - S10 show samples of these experimental results. The supplementary Movie S2. starts off with the first original source video frames and is followed by the simulation and experiment results shown side by side for the differentiator, integrator, and Hilbert transformer. This is then followed by the 34 functions (Supplementary Table S1) performed simultaneous by the massively parallel video processor. Finally, high order of derivatives are shown, and the video ends with results based on full 2 dimensional derivatives (see Methods).

3. Supplementary Note 3: Chromatic dispersion (CD) compensation

Chromatic dispersion (CD), which causes pulse broadening and limits the performance of the transversal structure, is one of the major impairments in the RF photonic system [s4]. In standard SMF, each optical frequency travels through the fibre at different velocities due to the CD properties of the fibre. It can be shown that the phase changes in the optical sidebands alter the resultant phase of the RF beat signals and the RF power p of the generated radio frequency f_{RF} will vary approximately as [s5]

$$p \propto (\cos\frac{1}{2}\beta_2\omega^2 z)^2 \tag{3}$$

where p is the RF power, ω is the RF angular frequency, z is the length of standard SMF, and β_2 is the group delay dispersion. Fig. S11 (a) shows the theoretical and measured second order dispersion involved power fading. In an approach, by using the phase modulating capabilities of the WaveShaper, which is capable of producing arbitrary group delay profiles of up to ±25 ps, it is possible to compensate for group delay ripple (GDR) and significantly improve the accuracy of the system. This compensation is achieved by configuring the WaveShaper to produce an equal but opposite GDR that counteracts the GDR of the system. By employing this approach, we successfully compensated the second-order dispersion (SOD) induced power fading and third-order dispersion (TOD) induced distortion of an integrator (Fig. S11 (b)). Fig. S11 (c) compares the processed images of 0.5 order differentiator with and without dispersion compensation method implemented. As can be seen in Fig. S11 (d), after implementing the dispersion compensation method, the RMSE of the processed signal has been improved from 0.0687 to 0.0558.

4. Supplementary Note 4: Theoretical speed of a scaled up system

Figure S12 shows the video image processor, using the C + L + S bands (with more than 405 wavelengths) and 19 spatial paths, and exploiting polarization. This would yield a speed of 64 GigaBaud × 8 bits/symbol × 2 × 81 × 19 = 1.575 Petabits/s. In this case the wavelengths would be distributed over 81 processors each have 5 taps in size (so that 81 × 5 = 405).

Finally, we discuss the potential energy efficiency of the optical signal processor. The power consumption of the comb source can be estimated as 1500 mW. The power consumption of the EDFAs can be estimated as 2000 mW (100 mW for each EDFA). The power consumption of the intensity modulator can be estimated as 3.4 V × 0.01 A = 34 mW. The overall computing speed of the optical signal processor is 2 × 34 × 5 × 62.9 = 21.386 TeraOPs/s. As such, the energy per bits of the optical signal processor can be roughly given as (1500 + 2000 + 34 × 19) mW / 21.386 TeraOPs/s = 0.194pJ/operation.

References of the Supplementary Materials

[s1] S. W. Huang et al., “Mode-locked Ultrashort pulse Generation from On-Chip Normal Dispersion Microresonators”, Physical Review Letters 114, 053901 (2015).
[s2] W. Wang et al., “Self-locked orthogonal polarized dual comb in a microresonator”, Photonic Research 6 (5), 363-367 (2021).
[s3] J. A. Davis, D. E. McNamara, and D. M. Cottrell, “Analysis of the fractional Hilbert transform”, Appl. Opt. 37 (29), 6911-6913 (1998).
[s4] A. Sheikh, C. Fougstedt, A. G. I. Amat, P. Johannisson, P. L. Edefors, and M. Karlsson, “Dispersion Compensation FIR Filter With Improved Robustness to Coefficient Quantization Errors,” Journal of Lightwave Technology 34 (22), 5110-5117 (2016).
[s5] G. H. Smith, D. Novak, Z. Ahmed, “Overcoming chromatic-dispersion effects in fiber-wireless system incorporating external modulators,” IEEE Transactions on Microwave Theory and Techniques 45 (8), 1410-1415 (1997).

Table S1. Detailed parameters for the massively parallel terahertz video image processor. DIFF (differentiation) HT (Hilbert transformation) BHT (Bandpass Hilbert transformation)

No.	Computing function	Order	Tap spacing (FSR)	SMF length (km)	Wave Shaper port	Tap coefficient	Tap number
1	DIFF	0.1	9	0.2	1	-0.1854, -0.1178, 1, -0.3339, -0.178	1,10,19,28,37
2	DIFF	0.2	9	0.2	2	-0.178, -0.0334, 1, -0.1697, -0.1478	2,11,20,29,38
3	DIFF	0.3	9	0.2	3	-0.1785, 0.0594, 1, -0.6135, -0.1088	3,12,21,30,39
4	DIFF	0.4	9	0.2	4	-0.1889, 0.1692, 1, -0.7738, -0.0591	4,13,22,31,40
5	DIFF	0.5	9	0.2	5	-0.2133, 0.3102, 1, -0.9645, 0.0054	5,14,23,32,41
6	DIFF	0.6	9	0.2	6	-0.2602, 0.5095, 1, -1.2127, 0.0929	6,15,24,33,42
7	DIFF	0.7	9	0.2	7	-0.3496, 0.8289, 1, -1.5802, 0.2236	7,16,25,34,43
8	DIFF	0.8	9	0.2	8	0.3737, 1, 0.6884, -1.5495, 0.3156	8,17,26,35,44
9	DIFF	0.9	9	0.2	9	-0.3472, 1, 0.303, -1.2558, 0.3344	9,18,27,36,45
10	HT	0.83	3	0.6	10	-0.2122, -0.6366, 0.2679, 0.6366, 0.2122	46,52,55,58,64
11	HT	0.88	3	0.6	11	-0.2122, -0.6366, 0.1763, 0.6366, 0.2122	47,53,56,59,65
12	HT	0.94	3	0.6	12	-0.2122, -0.6366, 0.0875, 0.6366, 0.2122	48,54,57,60,66
13	HT	1	3	0.6	13	-0.2122, -0.6366, 0, 0.6366, 0.2122	67,73,79,85
14	HT	0.78	3	0.6	14	-0.2122, -0.6366, 0.364, 0.6366, 0.2122	68,74,77,80,86
15	HT	0.72	3	0.6	15	-0.2122, -0.6366, 0.4663, 0.6366, 0.2122	69,75,78,81,87
16	DIFF	1.1	6	0.4	16	-0.3270, 1, -0.2479, -0.7689, 0.3165	70,76,82,88,94
17	Integration	1	1	5	17	1, 1, 1, 1, 1	89,90,91,92,93
18	BHT	1	5.5	0.4	18	0.2118, -0.3067, 0.3067, -0.2118	50,61,72,83
19	DIFF	1.0	11	0.15	19	-0.3333, 1, -1, 0.3333	51,62,84,95
20	DIFF	0.05	9	0.2	1	-0.1918, -0.1588, 1, -0.2671, -0.19	1,10,19,28,37
21	DIFF	0.15	9	0.2	2	-0.1808, -0.0762, 1, -0.4012, -0.1639	2,11,20,29,38
22	DIFF	0.25	9	0.2	3	-0.1772, 0.0115, 1, -0.5402, -0.1295	3,12,21,30,39
23	DIFF	0.35	9	0.2	4	-0.1823, 0.1115, 1, -0.6909, -0.0855	4,13,22,31,40
24	DIFF	0.45	9	0.2	5	-0.199, 0.2346, 1, -0.8641, -0.029	5,14,23,32,41
25	DIFF	0.55	9	0.2	6	-0.2331, 0.4, 1, -1.0789, 0.0454	6,15,24,33,42
26	DIFF	0.65	9	0.2	7	-0.2974, 0.6476, 1, d-1.3750, 0.1507	7,16,25,34,43
27	DIFF	0.75	9	0.2	8	-0.3942, 1, 0.9261, -1.7177, 0.2967	8,17,26,35,44
28	DIFF	0.85	9	0.2	9	-0.3585, 1, 0.483, -1.3967, 0.3278	9,18,27,36,45

29	HT	0.97	3	0.6	10	-0.2122, -0.6366, 0.0437, 0.6366, 0.2122	46,52,55,58,64
30	HT	0.92	3	0.6	11	-0.2122, -0.6366, 0.1317, 0.6366, 0.2122	47,53,56,59,65
31	HT	0.86	3	0.6	12	-0.2122, -0.6366, 0.2217, 0.6366, 0.2122	48,54,57,60,66
32	HT	0.81	3	0.6	14	-0.2122, -0.6366, 0.3153, 0.6366, 0.2122	68,74,77,80,86
33	HT	0.75	3	0.6	15	-0.2122, -0.6366, 0.4142, 0.6366, 0.2122	69,75,78,81,87
34	DIFF	0.95	6	0.4	16	-0.3391, 1, 0.1433, -1.1242, 0.3361	70,76,82,88,94

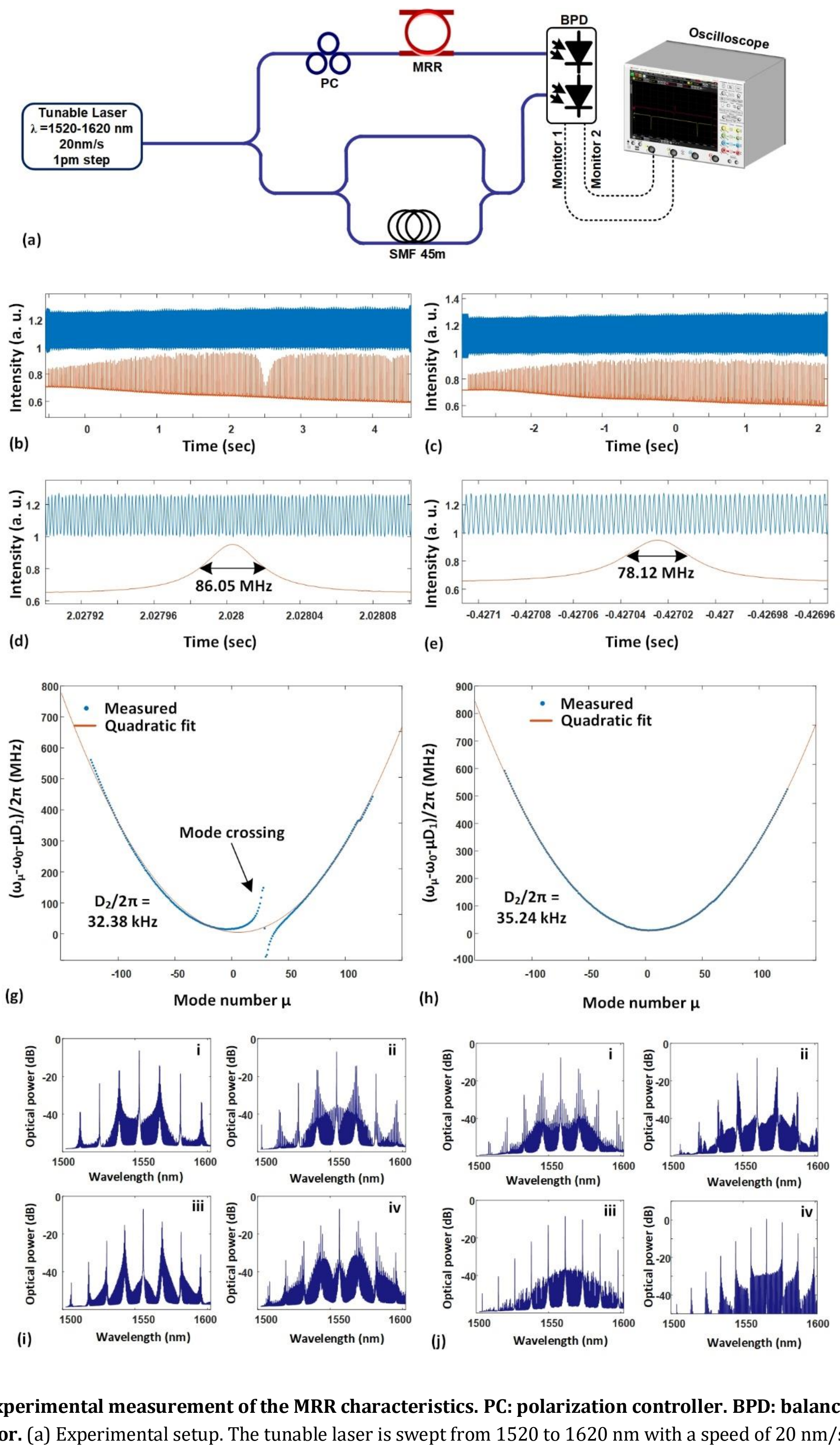

Figure S1. Experimental measurement of the MRR characteristics. PC: polarization controller. BPD: balanced photodetector. (a) Experimental setup. The tunable laser is swept from 1520 to 1620 nm with a speed of 20 nm/s in the measurements. SMF: single mode fibre with a length of 45 m. The transmission traces of TE mode and TM mode are measured

respectively by carefully tuning the PC. (b) and (c) The transmission traces of the TE and TM modes together with the fibre ring resonator transmission traces. (d) and (e) The FWHMs of the two vertically polarized modes are 86.05 MHz and 78.12 MHz, corresponding to Q factors of 2.2×10^6 and 2.4×10^6 respectively. (g) and (h) The calculated and measured dispersion curves of TE and TM modes. (i) and (j) The generated soliton crystal micro-combs of TE and TM modes, respectively.

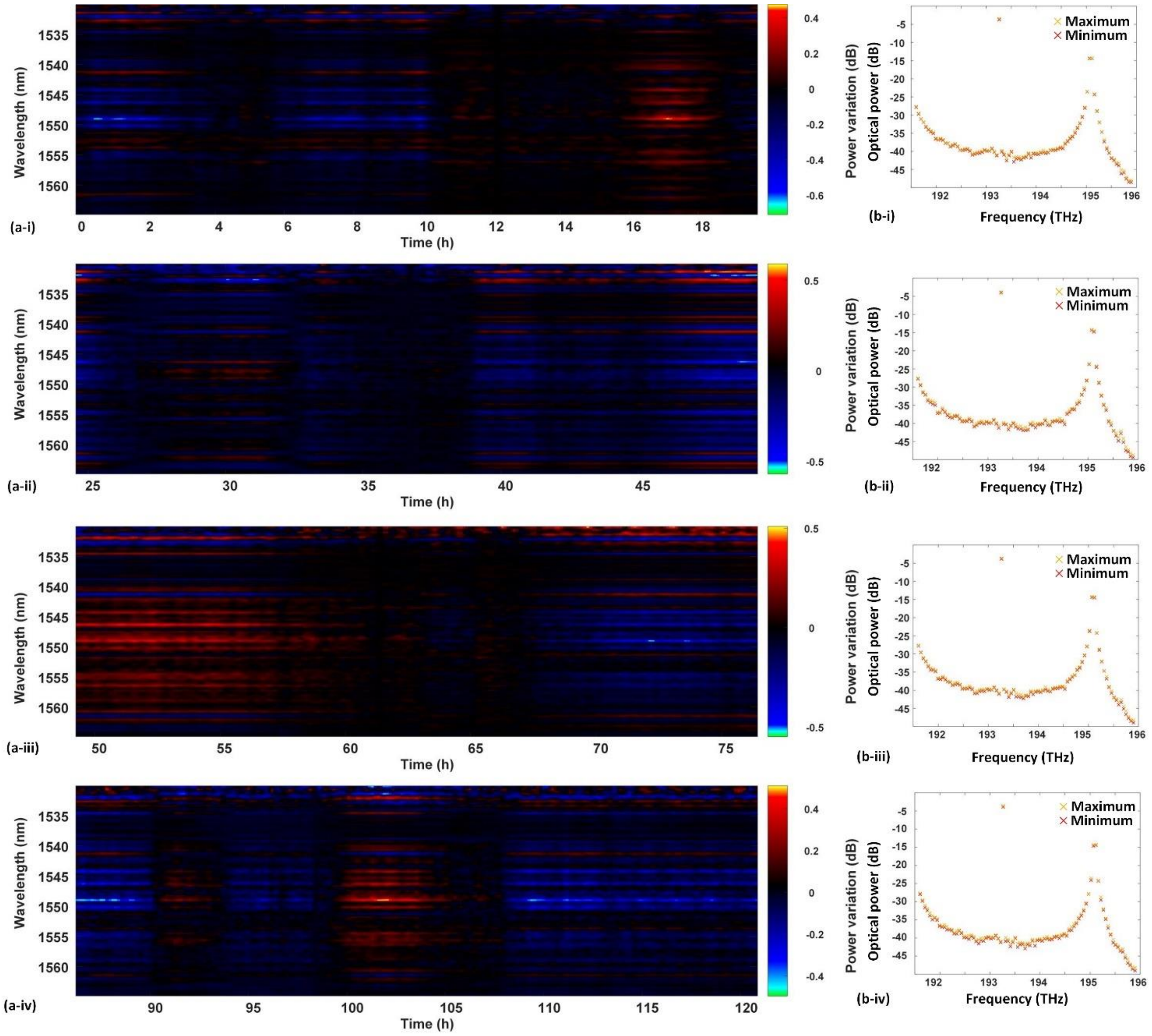

Figure S2. (a) Measured comb power stability over 120 hours (5 days) and extracted (b) deviation between the maximum and minimum optical power.

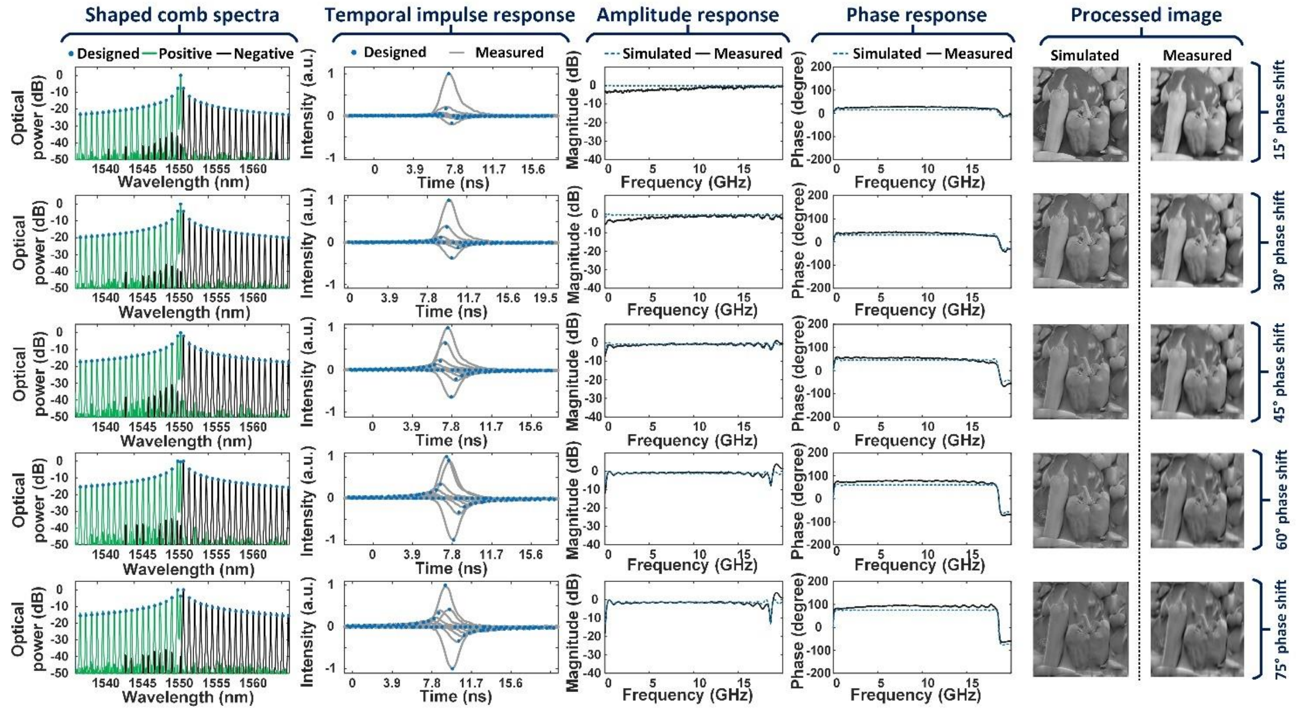

Figure S3. Simulated and measured shaped comb spectra, temporal impulse response, frequency response and processed image of fractional Hilbert transformer with tunable phase shift from 15° to 75°.

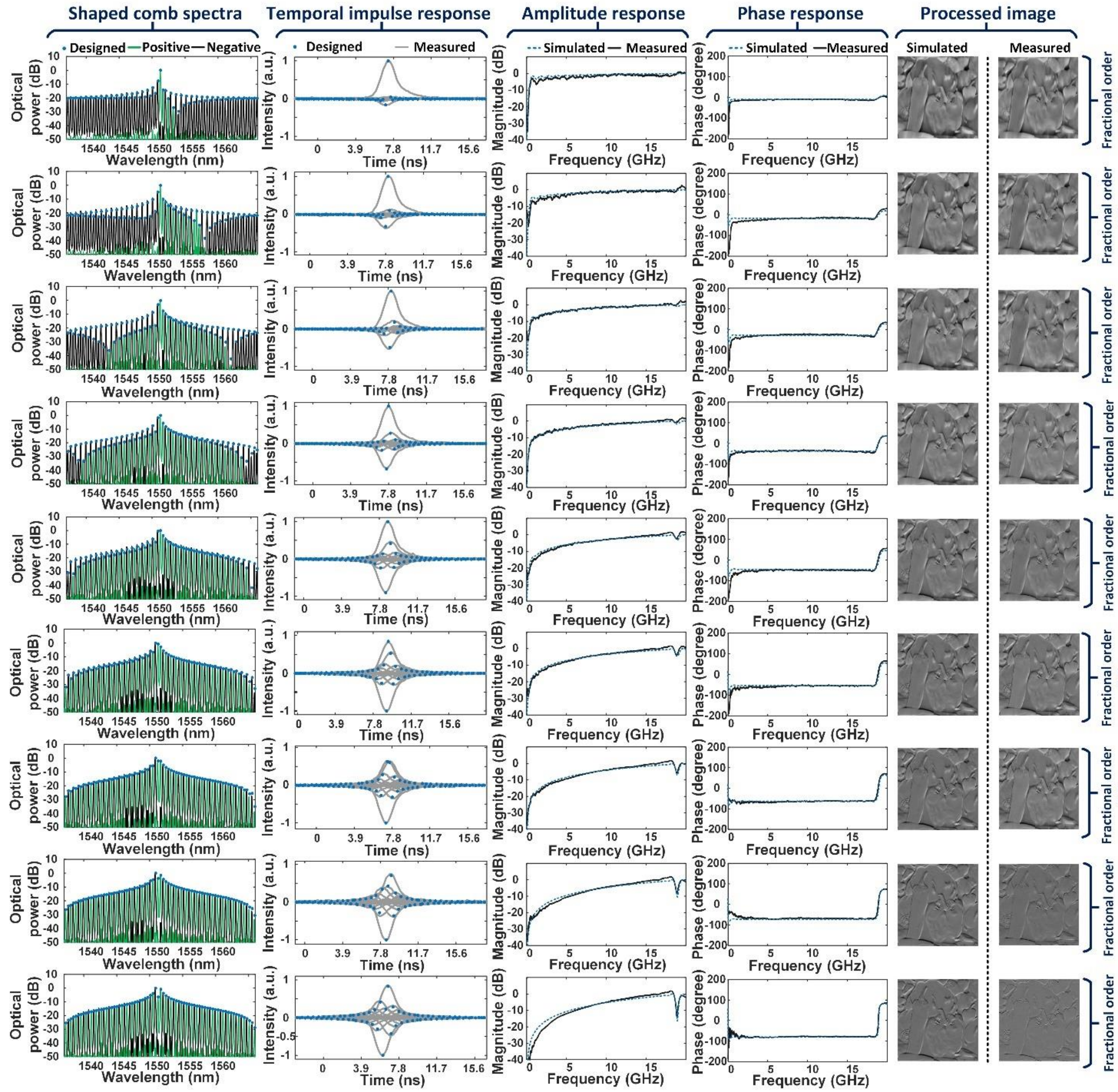

Figure S4. Simulated and measured shaped comb spectra, temporal impulse response, frequency response and processed image of fractional differentiator with tunable order from 0.1 to 0.9.

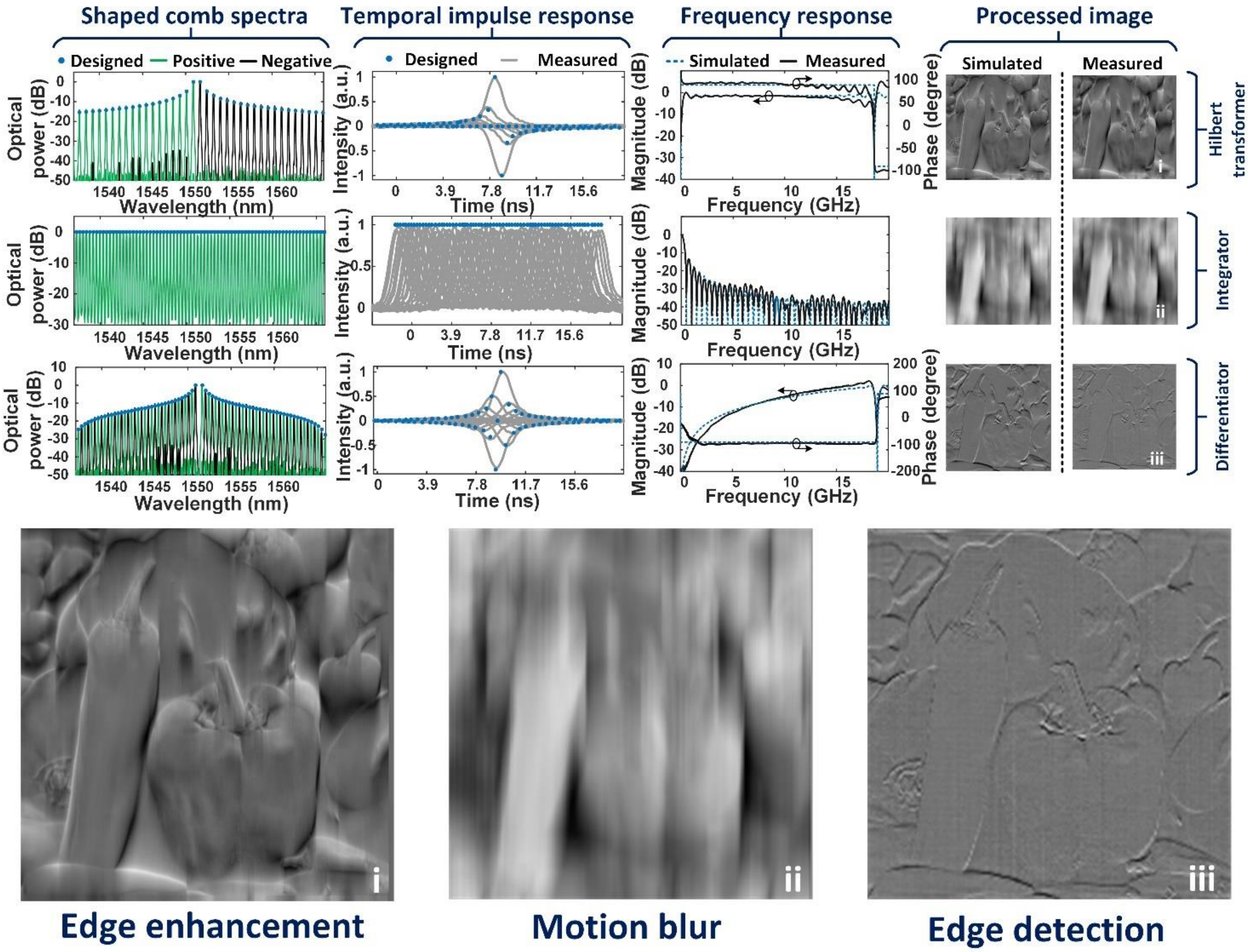

Figure S5. Simulated and measured shaped comb spectra, temporal impulse response, frequency response and processed image of integral signal processor include Hilbert transformer for (i) Edge enhancement, integrator for (ii) Motion blur, and differentiator for (iii) Edge detection.

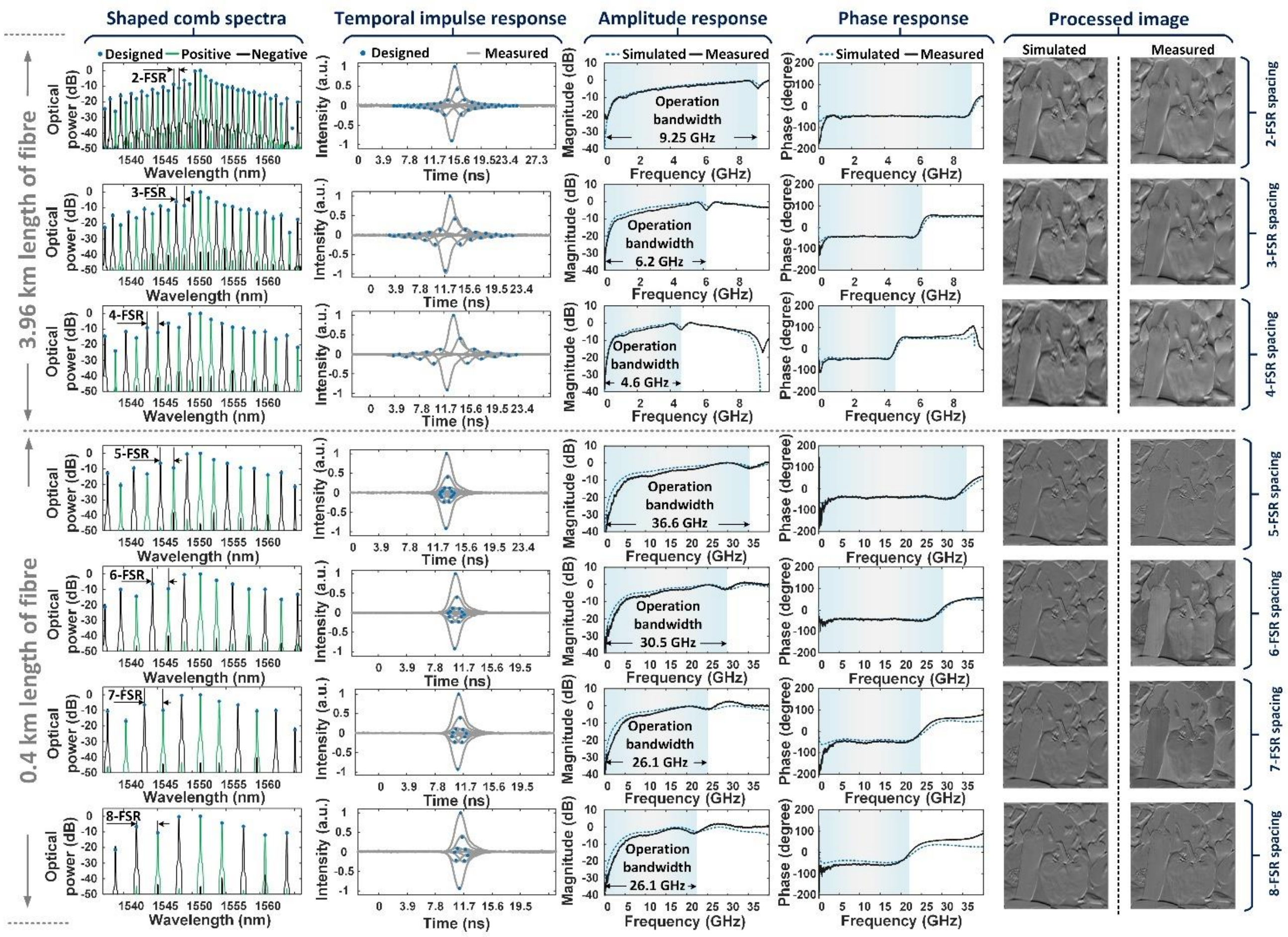

Figure S6. Simulated and measured shaped comb spectra, temporal impulse response, frequency response and processed image of 0.5 order fractional differentiator with varying comb spacing from 2-FSR spacing to 8-FSR spacing and with 0.4 km vs 3.96 km length of fibre.

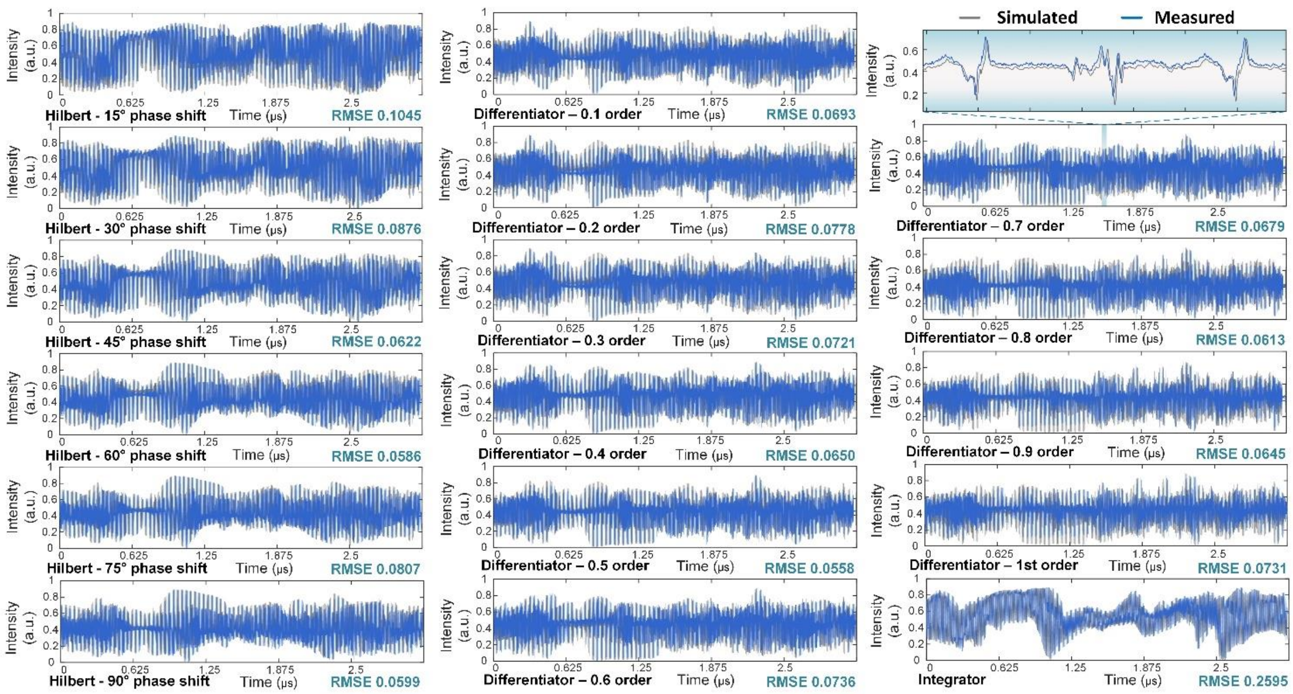

Figure S7. Simulated and measured processed waveforms as well as calculated RMSE.

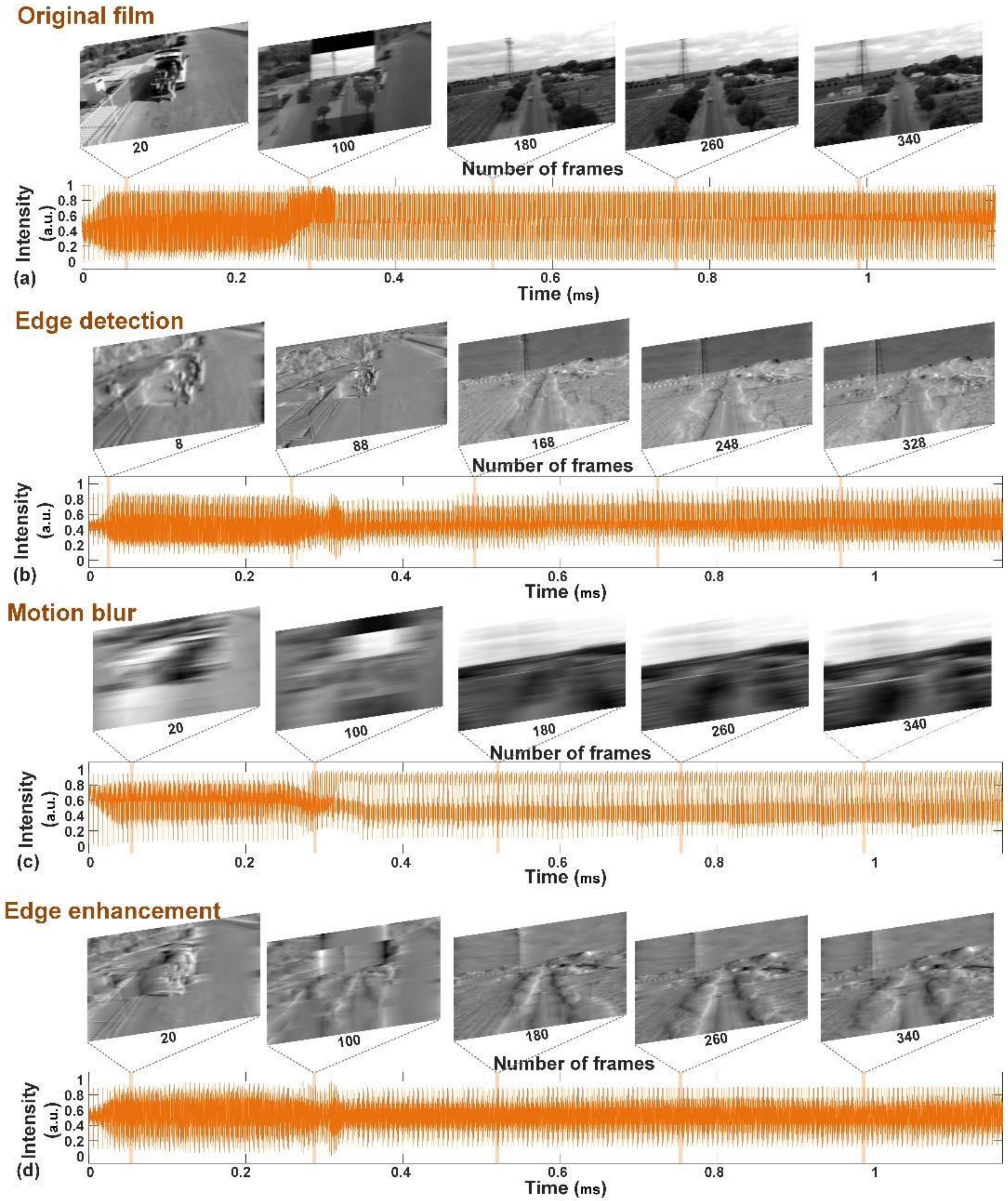

Figure S8. Extended data Figure 2. Measured video processing. (a) Original video. (b) Processed video after 0.5 order differentiation. (c) Processed video after integration. (d) Processed video after Hilbert transformation with 90-degree phase shift.

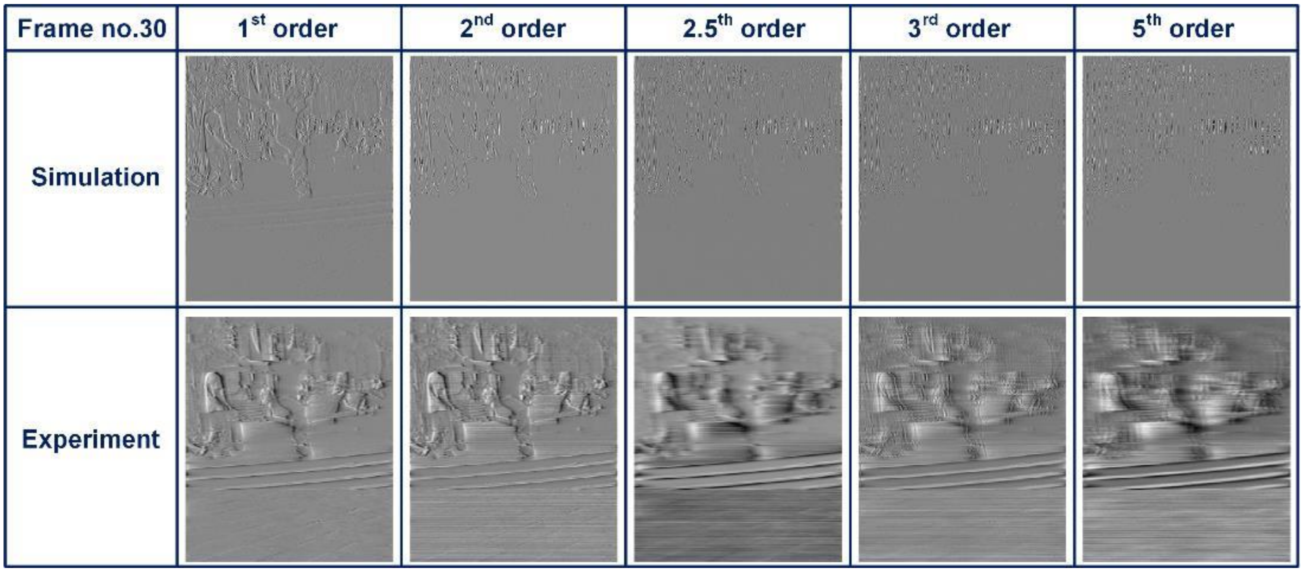

Figure S9. Extended data Figure 3. Simulated and measured higher order derivatives.

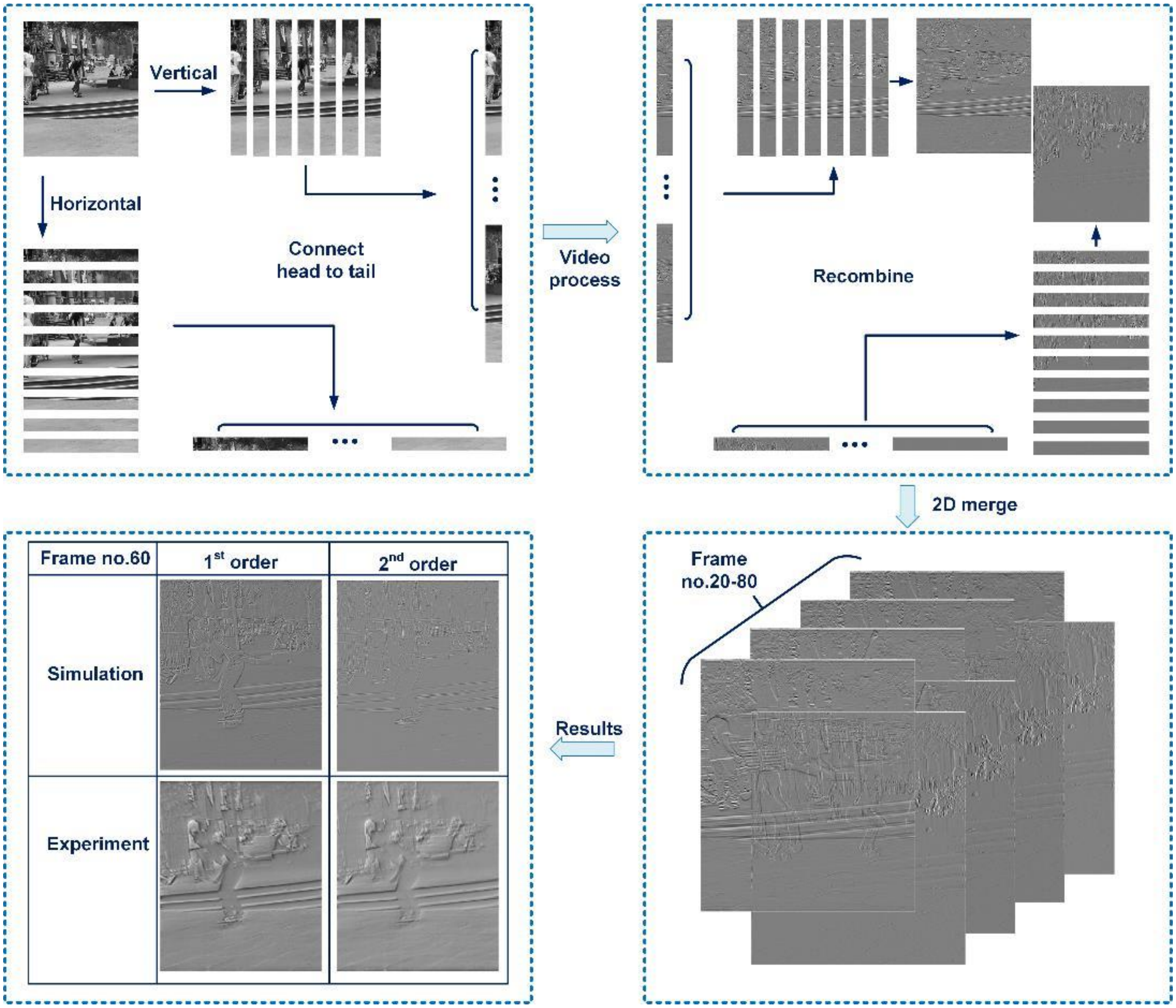

Figure S10. Extended data Figure 4. Illustration of two-dimensional derivative. Figure shows sequence of input data reorganization required for 2D derivatives – see Methods.

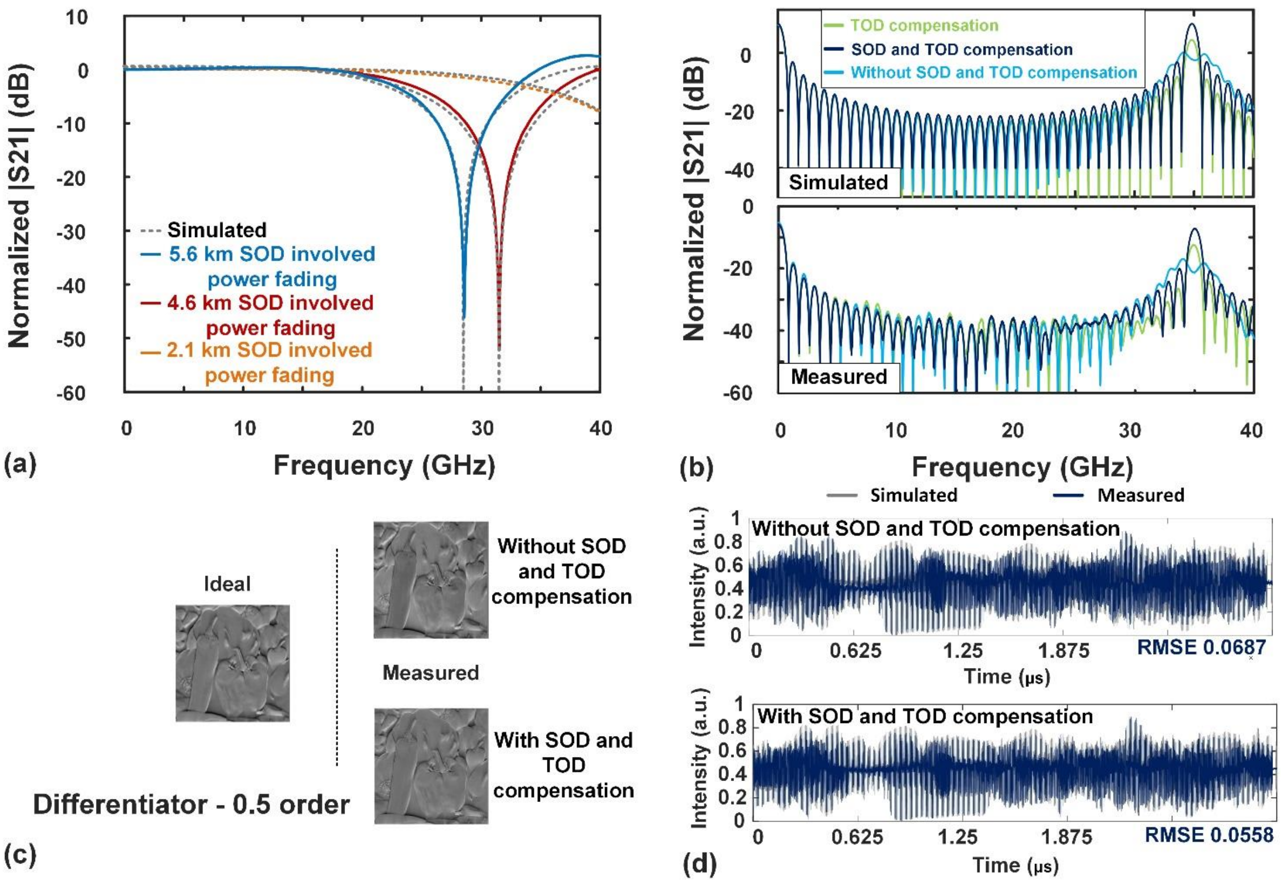

Figure S11. Simulated and measured (a) SOD involved power fading and (b) TOD involved distortion and an integrator with dispersion compensation method achieved. (c) processed image of 0.5 order fractional differentiator with and without SOD and TOD compensation method involved. (d) temporal results of 0.5 order fractional differentiator with and without SOD and TOD compensation method involved and RMSE of the results.

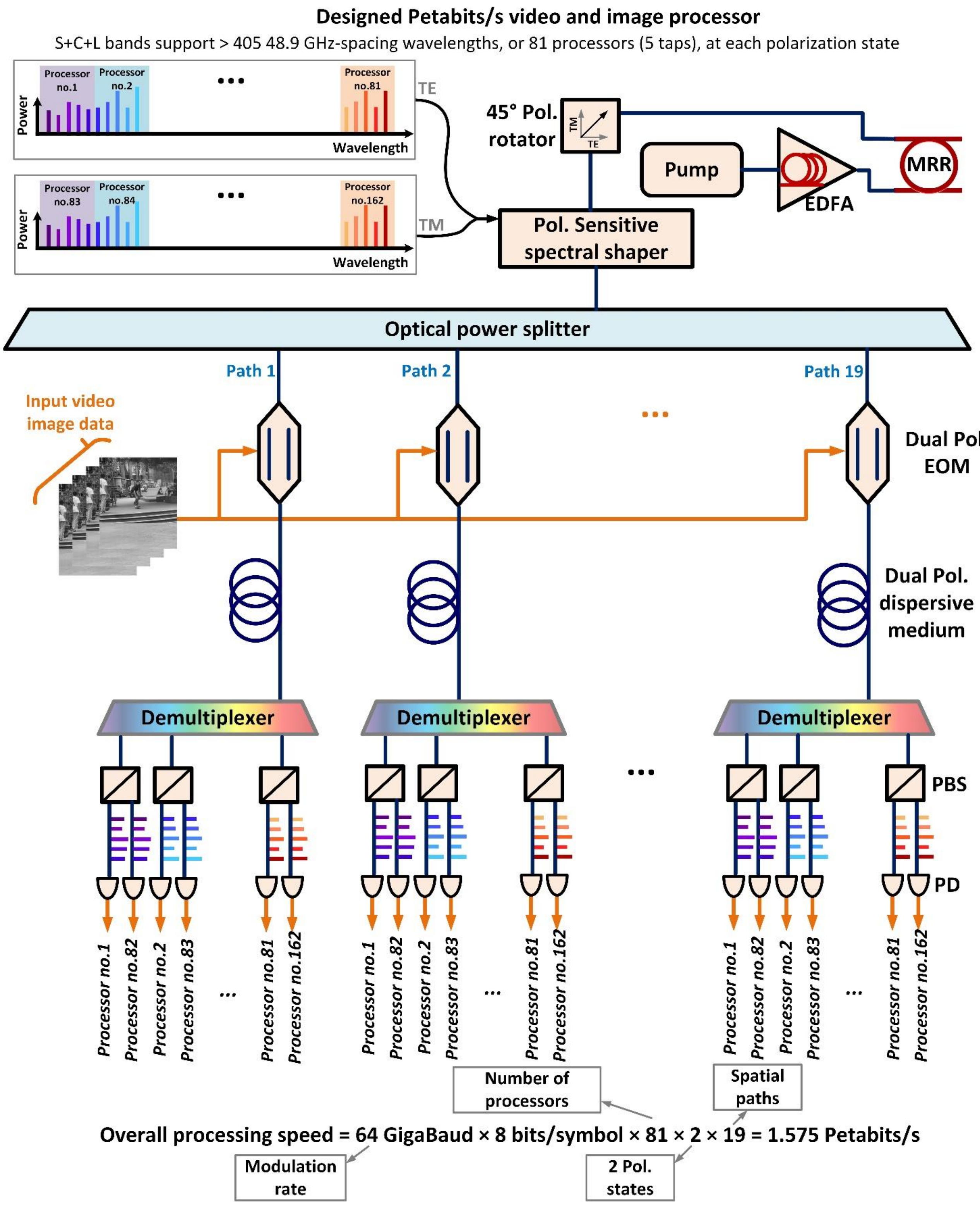

Figure S12. Designed scaled video image processor over the C + L + S bands, with spatial and polarization multiplexing. The 405 available wavelengths (on a 48.9 G grid) would be split into 81 processors each have 5 taps in size. Pump: continues-wave pump laser. EDFA: erbium doped fibre amplifier. MRR: micro-ring resonator. EOM: electro-optical Mach-Zehnder modulator. PBS: polarization beam splitter. PD: photodetector.

Table S2. Performance of achieved analog image processing functions.

	RMSE		RMSE		RMSE
Hilbert – 15° phase shift	0.1045	Differentiator – 0.1 order	0.0693	Differentiator – 0.7 order	0.0676
Hilbert – 30° phase shift	0.0876	Differentiator – 0.2 order	0.0778	Differentiator – 0.8 order	0.0613
Hilbert – 45° phase shift	0.0622	Differentiator – 0.3 order	0.0721	Differentiator – 0.9 order	0.0645
Hilbert – 60° phase shift	0.0586	Differentiator – 0.4 order	0.0650	Differentiator – 1.0 order	0.0731
Hilbert – 75° phase shift	0.0807	Differentiator – 0.5 order	0.0558	Integrator	0.2595
Hilbert – 90° phase shift	0.0599	Differentiator – 0.6 order	0.0736		
