# Supplementary material for: Photonic signal processor based on a Kerr microcomb for real-time video image processing
Source: Commun Eng. 2023 Dec 20;2:94. doi: 10.1038/s44172-023-00135-7 (PMC10956003; doi:10.1038/s44172-023-00135-7)
Supplement: Supplementary file 2 — Description of Additional Supplementary Files [file 44172_2023_135_MOESM2_ESM.pdf]

Description of Additional Supplementary Files

File name: Supplementary Movie S1

Description: Comb Generation

File name: Supplementary Movie S2

Description: Signal Processing Video
